# Rebound Acid Hypersecretion after Withdrawal of Long-Term Proton Pump Inhibitor (PPI) Treatment—Are PPIs Addictive?

**DOI:** 10.3390/ijms25105459

**Published:** 2024-05-17

**Authors:** Ken Namikawa, Einar Stefan Björnsson

**Affiliations:** 1 Division of Gastroenterology and Hepatology, Department of Internal Medicine, Landspitali University Hospital, 101 Reykjavik, Iceland; ken@landspitali.is; 2 Department of Gastroenterology, Cancer Institute Hospital, Japanese Foundation for Cancer Research, Tokyo 135-8550, Japan; 3 Faculty of Medicine, University of Iceland, 101 Reykjavik, Iceland

**Keywords:** proton pump inhibitors, rebound acid hypersecretion, withdrawal, discontinuation, gastrin

## Abstract

Proton pump inhibitors (PPIs) are widely used in the long-term treatment of gastroesophageal reflux disease (GERD) and other upper gastrointestinal disorders, such as the healing of peptic ulcers and/or prophylactic treatment of peptic ulcers. PPIs are also widely used as symptomatic treatment in patients with functional dyspepsia. One of the adverse effects of the long-term use of PPI is rebound acid hypersecretion (RAHS), which can occur after the withdrawal of PPI therapy due to a compensatory increase in gastric acid production. Mechanisms of the RAHS have been well established. Studies have shown that pentagastrin-stimulated acid secretion after the discontinuation of PPIs increased significantly compared to that before treatment. In healthy volunteers treated with PPIs, the latter induced gastrointestinal symptoms in 40–50% of subjects after the discontinuation of PPI therapy but after stopping the placebo. It is important for practicing physicians to be aware and understand the underlying mechanisms and inform patients about potential RAHS before discontinuing PPIs in order to avoid continuing unnecessary PPI therapy. This is important because RAHS may lead patients to reuptake PPIs as symptoms are incorrectly thought to originate from the recurrence of underlying conditions, such as GERD. Mechanisms of RAHS have been well established; however, clinical implications and the risk factors for RAHS are not fully understood. Further research is needed to facilitate appropriate management of RAHS in the future.

## 1. Introduction

Proton pump inhibitors (PPIs) are widely used in the long-term treatment of refractory symptoms associated with gastroesophageal reflux disease (GERD) and various other gastrointestinal conditions. PPI use has increased worldwide since its development in the late 1980s [1,2].

Meanwhile, long-term suppression of gastric acid secretion by PPIs can cause a variety of adverse effects, such as changes in intestinal microbiota, malabsorption of nutrients, and changes in metabolic pathways [3,4,5,6,7,8].

Recent studies have also reported that the long-term use of PPIs is associated with an increased risk of gastric cancer in patients compared to those without PPI therapy [9,10,11]. Therefore, unnecessary long-term prescriptions should be avoided. However, specific problems can occur in the discontinuation process of PPIs. Abrupt discontinuation of PPIs can increase gastric acid production above pre-PPI treatment levels, which are related to persistent hypergastrinemia, secondary to inhibition of acid secretion by using PPIs. This phenomenon’s biological mechanism and pathophysiology have been established in both animal and human research studies and have been defined as rebound acid hypersecretion (RAHS) [11,12,13].

Some well-designed studies have been conducted to determine the role of RAHS in clinical settings [13,14,15,16]. In a study from Norway, Waldum et al. [13] measured basal and pentagastrin-stimulated acid secretion in patients with GERD before and 14 days after the end of a 3-month treatment period. A significant increase in basal secretion and a marked (50%) and significant increase in pentagastrin-stimulated acid secretion were found after PPI treatment resulted in RAHS. Gillen et al. [14] conducted a study in the UK to examine gastrin and intragastric pH before, on, and after PPI treatment in 12 *Helicobacter pylori* (*H. pylori*)-negative and 9 *H. pylori*-positive patients. Rebound acid hypersecretion (in both basal and maximal acid output) occurred in *H. pylori*-negative subjects after treatment. Apart from these physiological studies, two clinical randomized, double-blind, placebo-controlled trials on healthy volunteers conducted in Sweden, using 48 asymptomatic subjects [15], and Denmark, with 120 asymptomatic subjects [16], demonstrated that the subjects in both studies randomized to the PPI therapy developed symptoms associated with RAHS, whereas symptoms did not occur in those who were randomized to placebo.

The purpose of this review is to summarize the current knowledge in the field of RAHS, discuss its limitations, and guide future directions of research.

### 1.1. Incidence

In the two abovementioned randomized controlled trials (RCTs), asymptomatic healthy volunteers were randomized to a PPI, i.e., esomeprazole in one study [16] and pantoprazole in the other study [15], or a placebo group. Interestingly, despite some differences in design and a different PPI type, the same proportion of subjects (44%) developed acid-related symptoms after the discontinuation of PPI therapy, which were significantly higher than those of the control group with placebo.

In patients with endoscopy-negative reflux disease, no aggravation of symptoms was found after on-demand use of PPI; however, 32% of patients experienced increased symptom burden [17]. A recent study in Japan showed that symptoms of GERD appeared in 27.5% of patients after the discontinuation of 4 weeks of antacid treatment (PPI or vonoprazan) [18].

Regarding the physiological study undertaken in the UK, basal acid output increased in approximately 83% of the subjects and maximal acid output increased in 100% of the subjects at day 15 after the discontinuation in *H. pylori*-negative patients however, the degree of increase varied among individuals [14].

### 1.2. Symptoms and Their Severity

In the clinical studies on healthy volunteers, symptoms of RAHS were generally mild to moderate and mainly consisted of heartburn and regurgitation, but symptoms also included non-specific dyspepsia [15,16]. In the study by Reimer et al. [16], the symptoms and severity of RAHS were assessed by disease-specific questionnaires using the Gastrointestinal Symptom Rating Scale [19,20]. The Glasgow Dyspepsia Questionnaire [21] was used in the study by Niklasson et al. [15] and Juul-Hansen et al. [17]. Farup et al. adopted the Visual Analog Scale for assessment [22].

Heartburn or regurgitation was more frequently reported in 77% (20/26) of the subjects compared to dyspepsia in 42% (11/26) of the subjects [16]. Gillen et al. showed that the severity of RAHS was related to the degree of elevation in pH in the stomach during PPI treatment [14].

### 1.3. Onset and Duration of Symptoms

Symptoms after the discontinuation of PPI therapy in previously asymptomatic subjects appeared at day 5–14 post withdrawal in the majority of subjects and lasted 4–5 days on average [15,16], and in one of the studies, 38% had onset of symptoms at week 3–4 post withdrawal [16]. Considering these data, 9 days and 14 days of follow-up adopted in other studies [17,22] may be too short to capture all the symptoms potentially related to RAHS, which could lead to the result of no rebound aggravation.

Regarding the duration of rebound acid secretion, after more than one year of treatment with PPIs, Fossmark et al. found that rebound hypersecretion lasted more than 8 weeks but less than 26 weeks. In this study, basal and pentagastrin-stimulated acid output, chromogranin (CgA), and gastrin were measured at 4, 8, 16, and 26 weeks. Pentagastrin-stimulated acid secretion was higher at 4 and 8 weeks than at 26 weeks after PPI discontinuation. Gastrin and CgA were significantly reduced at 4 and 8 weeks, respectively [23]. Boyce et al. showed that increased gastrin and CgA by week 4 of PPI use returned to the baseline within 2–3 days from withdrawal in their randomized double-blind study using 48 subjects [24]. Juul-Hansen et al. reported that significantly increased p-CgA and s-gastrin after 6 months of on-demand PPI treatment returned to pretreatment levels 14 days after withdrawal [17].

### 1.4. Duration of PPI Treatment

In the two aforementioned RCT studies, undertaken in Sweden and Denmark [15,16], acid-related symptoms were identified after PPI use for 4 and 8 weeks, whereas no study adopting PPI use within 2 weeks demonstrated symptoms suspected of RAHS. One study used a treatment period of only 5 days, which was not able to detect symptoms of RAHS [22]. Thus, the short duration of PPI therapy is probably inadequate to induce physiological RAHS. In another study, 24-h ambulatory esophageal pH measurements were performed, and the results suggested that PPI therapy for 1 week did not cause acid rebound, although 3 days of follow-up period in this study might be inadequate for precise evaluation [25]. Peura et al. [26] reported that a 14-day regimen of PPIs for frequent heartburn did not cause any symptomatic rebound during 1-week follow-up periods. Boyce et al. [24] also reported similar results; gastrin and CgA increased by 4-week PPI; however, no rebound dyspepsia was observed after withdrawal, which was consistent with the results that increased gastrin and CgA returned to the baseline within 2–3 days from withdrawal. Another study using on-demand PPI treatment (mean of 15.1 mg/day of lansoprazole) for 120 days [17] did not show aggravation of symptoms. Although s-gastrin and p-CgA increased after treatment, this study was not designed to assess RAHS. Currently, there is insufficient evidence to conclude that on-demand PPI therapy does not lead to physiological RAHS.

In terms of the duration of the abovementioned physiological studies, which demonstrated RAHS, 90-day [13] and 8-week PPI therapy [14] were adopted.

It is conceivable that RAHS might be affected by the duration of PPI treatment. On the other hand, in a recent study on 100 PPI users on long-term therapy and 50 healthy volunteers as controls, no significant correlation was found between the area under the gastrin curve measured after a meal and the PPI treatment duration. In this study, PPI duration was classified into three subgroups depending on the duration of exposure: 2–5 years, 5–10 years, and >10 years [27].

### 1.5. Physiological Mechanisms

**Figure 1:**
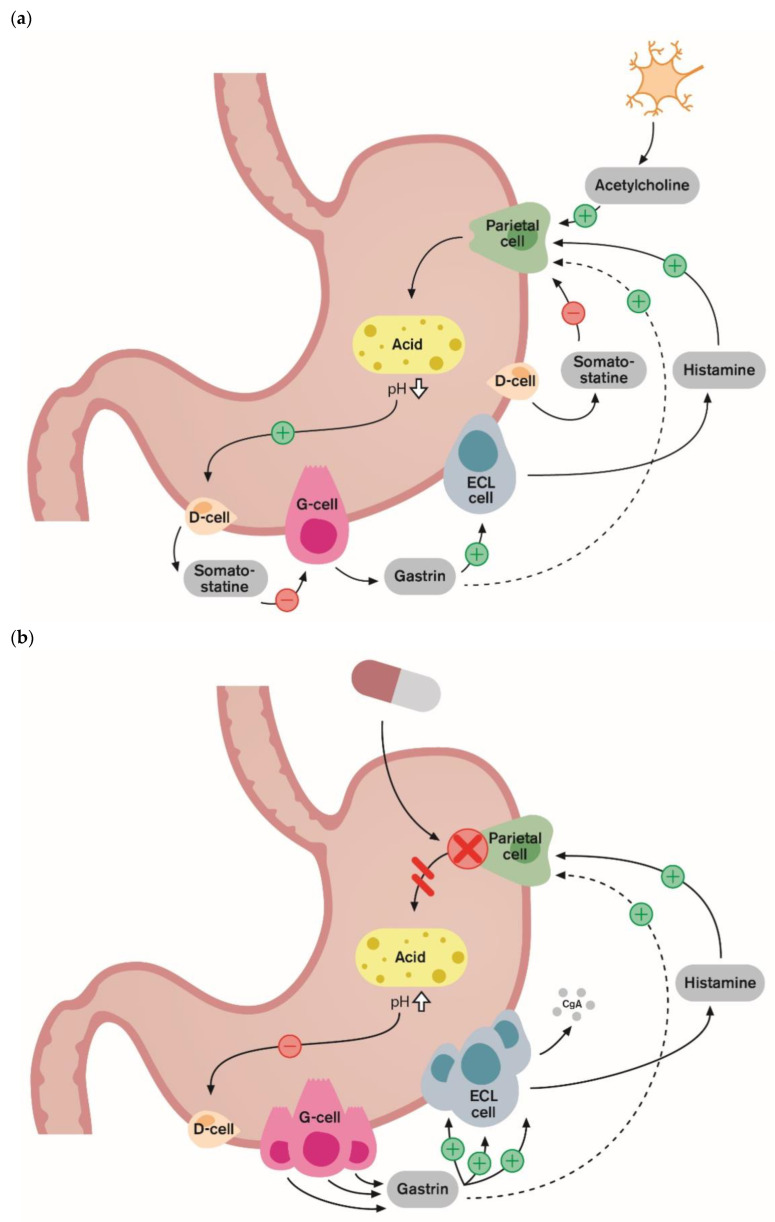

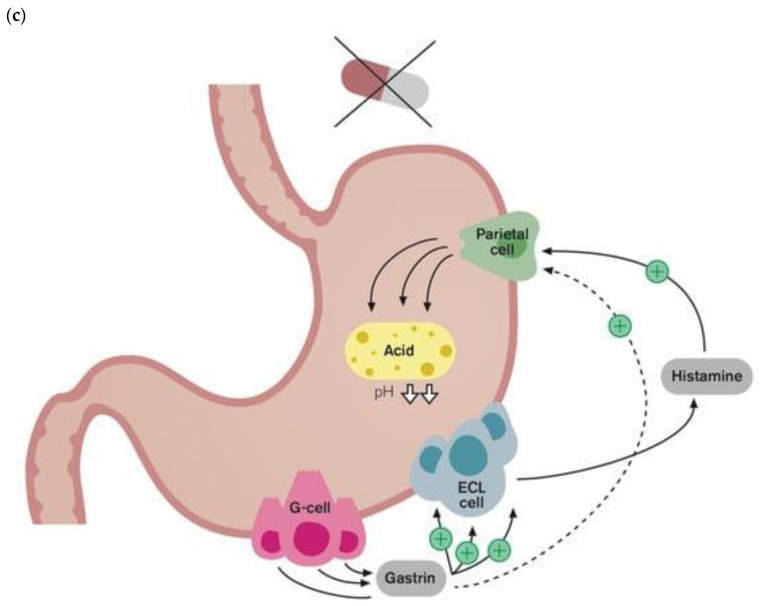
Physiological mechanisms of rebound acid hypersecretion: (**a**) under normal physiological conditions, protein in meals stimulates the G-cells to release gastrin into the blood. Gastrin stimulates the enterochromaffin-like (ECL) cells to release histamine. The histamine then stimulates acid-producing parietal cells. This is the gastrin–ECL axis, the main stimulatory pathway of gastric acid secretion. The over-production of acid is prevented by negative feedback inhibition by intragastric acidity as low antral pH inhibits gastrin release via somatostatin from D-cells. (**b**) Protein pump inhibitors (PPIs) inhibit gastric acid secretion by binding covalently to active proton pumps on the parietal cells. This prevents acid secretion and leads to hypoacidity (higher pH level). Thus, somatostatin-mediated negative feedback of gastrin release on antral G-cells is inhibited, which leads to hypergastrinemia, and gastrin exerts a trophic effect on gastric mucosa, causing enterochromaffin-like (ECL) hyperplasia. (**c**) The measurement of CgA levels in the blood can be a useful tool for monitoring ECL cell hyperplasia secondary to treatment with PPIs; following PPI discontinuation, the recovery of acid secretion can be exaggerated. Hypergastrinemia secondary to PPI therapy is associated with acid hypersecretion or the so-called rebound acid hypersecretion phenomenon. ECL cell: enterochromaffin-like cell. These figures were adapted from Helgadottir 2009 [29].

The biological mechanism and pathophysiology of RAHS have been well established [11,12,13,14]. Ultimately, the role of PPI therapy is to decrease acid secretion in the stomach. PPIs are absorbed in the proximal small bowel, and once in circulation, they affect the parietal cells of the stomach. This drug binds to and inhibits the function of the H+/K+ ATPase enzyme (also known as the gastric proton pump), which is responsible for the transport of hydrogen ions into the gastric lumen [28]. Figure 1 summarizes the mechanisms of acid production under normal physiological conditions, responses under chronic inhibition of acid secretion by PPIs, and acid rebound by the discontinuation of PPIs.

Gastrin is a peptide hormone, which, in the case of lowered acid secretion or increased pH in the stomach, triggers the secretion of gastric acid by parietal cells through the activation of enterochromaffin-like (ECL) cells. Gastrin is released from antral G-cells, stimulating (ECL) cells via gastrin/cholecystokinin2 (CCK2) receptors accelerating histamine release. Then, histamine stimulates parietal cells to produce acid. Under normal conditions, gastrin participates in negative feedback regulation that involves acid-induced release of somatostatin from the antral D cell [30]. Reduced gastric acidity under chronic inhibition by long-term PPI use leads to elevated serum gastrin levels, resulting from a dysregulated negative feedback system via antral G-cell hyperplasia. Hypergastrinemia is a compensatory response that causes ECL cell hyperplasia, stimulating histamine secretion. This PPI-induced gastrin elevation is thought to play a role in rebound hyperacidity when PPIs are discontinued. Stimulation of parietal cell acid secretion by increased histamine through hypergastrinemia is still interrupted during PPI therapy. However, once PPIs are discontinued, the recovery of acid with elevated secretion capacity can be exaggerated. This mechanism was well demonstrated in the study by Waldum et al. [13]. Gastrin and CgA increased during PPI treatment in patients with GERD, and both basal and pentagastrin-stimulated acid secretion after the discontinuation of PPIs also increased significantly compared to those before treatment. In a study from Scotland, Gillen et al. aimed to study the effects of omeprazole on gastrin-stimulated acid secretion, investigate the role of *H. pylori*, and find out if post-treatment rebound acid secretion was related to the degree of acid suppression and elevation of gastrin levels (14). Gillen et al. [14] showed that both basal and maximal gastric acid output after the discontinuation of PPI therapy were higher than before PPI treatment, and its severity was related to the degree of elevation of both pH and gastrin levels on treatment. Regarding the symptoms of RAHS, gastrin and p-CgA, which correlate positively with serum gastrin, were shown to be significantly correlated to acid-related symptoms during PPI treatment and after discontinuation [15,16]. In these studies, RAHS was only indirectly assessed by changes in gastrin or CgA levels, which indicate gastric acid secretory capacity; in other words, direct measurements of the amount of gastric acid secretion or acid reflux in the esophagus were not performed. However, these findings were consistent with the role of RAHS in the symptoms of post-PPI withdrawal. In one study, gastroesophageal reflux was measured by 24 h of pH monitoring and gastric acid secretory capacity before and after PPI treatment [17]. However, no differences were found with regard to time with pH < 4, suggesting that RAHS does not induce gastroesophageal reflux. These results might be explained by the insufficient consumption of PPIs on demand, suggested by the return of CgA and gastrin to pretreatment levels 14 days after withdrawal in this study.

### 1.6. Risk Factors

The risk factors for RAHS are not fully understood. One of the risk factors is a negative *H. pylori* status. The relationship between *H. pylori* status and the development of RAHS is not clear due to the lack of studies on this relationship. Niklasson et al. [15] found symptoms of RAHS from *H. pylori*-negative subjects randomized to PPI therapy in an RCT with healthy volunteers. However, that study did not include a control group of *H. pylori*-positive subjects in order to compare RAHS in patients with and without *H. pylori* infection. In another RCT study, subgroup analyses regarding *H. pylori* status were not conducted because the majority of the infected subjects (88.9%; 8/9) were randomly allocated to placebo [16]. Farup et al. [22] reported that the results did not significantly differ between *H. pylori* -positive and -negative patients; however, there was an imbalance in subjects based on *H. pylori* status; *H. pylori*-positive patients accounted for only 21% (13/62). Considering the mechanism of RAHS, it seems clear that acid rebound occurs more frequently in *H. pylori*-negative subjects with more gastric acid secretory capacity than in *H. pylori*-positive subjects. Previous studies have revealed a strong association between *H. pylori* infection and gastric acid secretory capacity [31,32]. Gillen et al. [14] found RAHS in *H. pylori*-negative subjects but not in *H. pylori*-positive subjects. Wada et al. [33] examined the clinical necessity of acid inhibitors to prevent GERD or reflux esophagitis caused by RAHS in RCT with 39 patients who underwent successful *H. pylori* eradication. After 8 weeks of PPI treatment and following 16 weeks of treatment with a preventive drug, reflux esophagitis assessed by endoscopy and GERD assessed by a symptom-related score were compared to those of the baseline. In total, only two patients demonstrated rebound symptoms (one patient) or reflux esophagitis (one patient). In this report, the authors concluded that there was little necessity to use preventive drugs for acid rebound by PPI discontinuation in patients after *H. pylori* eradication with severe gastric atrophy, who had lost their main acid secretory capacity.

Only a few studies have aimed to investigate the impact of gender on PPI treatment, and some of the findings on gender differences indicate that females might be more sensitive to PPIs’ inhibitory effects on acid secretion and that they induced more gastrin release compared to males [33,34,35,36]. Helgadóttir et al. conducted a double-blind RCT in Iceland using 100 patients (including 49 females) with endoscopically verified erosive esophagitis on long-term PPI therapy to investigate gender differences in reducing the dose of PPI. The patients were subclassified into a step-down group with their dose being reduced by half during treatment or into a group with the same dose for 8 weeks. The rates of successful reduction in PPI, symptom severity, and s-gastrin level were evaluated. Female patients showed higher gastrin levels compared to male patients: 78 pg/mL (IQR, 50 to 99) versus 50 pg/mL (IQR, 36 to 74) (*p* = 0.007). Female patients on long-term PPI therapy were three times more likely to tolerate half of their prior dose, and they demonstrated a higher probability for successful step-down: among those randomized to the step-down intervention, only 3/25 (12%) women failed to complete 2 months of lower-dose therapy, compared to 9/25 (36%) men (*p* = 0.09). These results indicate that females with gastroesophageal reflux disease might tolerate lower doses of PPIs compared to males [34]. However, the impact of the female gender on the development of RAHS has not been studied.

Regarding other risk factors, Tanaka et al. [18] conducted a multicenter study in Japan using 96 patients with erosive GERD. They examined the relationship of patient back-grounds (gender, age, body mass index, alcohol consumption, and smoking habits), hiatal hernia, *H. pylori* infection, pepsinogen I and II concentrations and I/II ratios, and s-gastrin levels with a scale for GERD symptoms before and after drug discontinuation. In this study, no related risk factors for rebound were detected.

### 1.7. Types and Doses of PPIs

Several types of PPIs have been used in the studies that demonstrated symptoms and/or physiology of RAHS: esomeprazole 40 mg [16], pantoprazole 40 mg [15], and omeprazole 40 mg [14]. On the other hand, the following combinations of PPIs and doses reportedly did not cause RAHS: esomeprazole 20 mg (14 days of PPI use and a 1-week follow-up period [26] and 7 days of PPI use and a 3-day follow-up period [25]) and lansoprazole 60 mg (5 days of PPI use and 9 days of follow-up) [22]. The design, methods, and outcome of the studies that investigated the physiology and symptoms of RAHS associated with PPI therapy are summarized in Table 1.

It seems that RAHS is an adverse effect related to the class of PPIs and is not related to the type of PPIs but rather related to the magnitude of gastric acid inhibition. A study with on-demand PPI use demonstrated a positive correlation between total consumption of lansoprazole and CgA increase during treatment [17].

The discontinuation of H2-receptor antagonist (H2RA) has also been shown to lead to RAHS [21,37,38,39]. Smith et al. found symptomatic rebound with a median onset and a median duration of symptoms for 2 days following treatment with H2RA [37]. Compared to PPIs, the shorter periods before onset and lasting symptoms seen in post-withdrawal H2RA therapy both seem physiologically well-explained, considering the mechanism of reversible binding to the H2 receptor and the less profound acid inhibition induced by this type of medicine.

In a recent study, the discontinuation of vonoprazan, which is a member of a new class of acid suppressants (potassium-competitive acid blockers [40]), was also associated with symptoms thought to be due to acid rebound [18]. In this study, the severity and frequency of reflux symptoms after discontinuation in the vonoprazan group tended to be higher than in the PPI group. The gastrin level at week 4 from withdrawal in the vonoprazan group was also higher than that of the PPI group. Considering the greater demonstrated acid inhibitory effect than PPIs [41,42], these results are not surprising.

### 1.8. Prevention and Management

Tapering of PPI therapy has been suggested as a way of avoiding the potential consequences of RAHS. Thus, some researchers have recommended tapering (dose or on-demand use) in order to decrease the risk of acid rebound based on the results demonstrating its efficiency for the control of symptoms or successful discontinuation of PPIs [43,44,45,46]. However, there is a lack of evidence to demonstrate its efficiency. Hendricks et al. [47] recently conducted an RCT in the USA with 38 GERD patients to investigate the difference in the successful discontinuation of PPI use after 12 months between patients discontinuing it abruptly or by tapering it. Although there was no significant difference between the two groups, there were fewer symptoms associated with the tapering method. On the other hand, in the RCT study by Björnsson et al. [48], no difference in the fraction of patients that restarted PPI after 6 months was found between the patients with abrupt withdrawal or the patients with tapering over three weeks. It is conceivable that a longer duration of tapering might be helpful, but evidence of this method is lacking. To address this lack of evidence for tapering methods, further organized studies are needed. The RCT by Hojo et al., a multicenter study in Japan, is currently ongoing to investigate the efficiency of tapering methods with 90 GERD patients, randomized to a group with abrupt PPI discontinuation or a group with gradual dose tapering [49]. In this study, the proportion of patients who successfully discontinued the PPI will be evaluated as primary outcomes 6 months and 12 months after the start of the study. Additionally, the symptoms will also be assessed at five timepoints; the start of the study, 2 weeks, 4 weeks, 6 months, and 12 months after the start of the study.

In recent years, some attempts have been made to explore effective ways for discontinuing PPIs to prevent acid rebound. In the abovementioned RCT by Hendricks et al. [47], H2 blocker use was associated with the successful discontinuation of PPIs. The use of alginate, which suppresses reflux symptoms by forming a physical barrier that protects the delicate esophageal mucosa [50,51], was reportedly useful in preventing symptom exacerbation during pre-investigation PPI wash-out [52,53]. In the study conducted in the UK by Vales et al. [53], 48 patients after ≥4 weeks of PPI therapy were randomized to alginate use or the control group. While patients in the control group had a significant increase in symptoms (median difference: 6.5, 95% CI (1 to 7), *p* = 0.04), no change occurred in the group using alginate (median difference −1.5, 95% CI (−2, 3.5), *p* = 0.54). Coyle et al. [52] demonstrated that the combination of education program and management with alginate for rebound symptoms helped the successful step-down or discontinuation of PPIs. In this prospective interventional study, trained nurse advisers gave a 20-min educational lecture on the management of PPIs for de-prescription, including acid rebound, and alginate was supplied for the self-management of rebound symptoms. After 12 months, 75% of 6249 eligible patients stepped down or off PPIs (35.3% stepped off; 5.0% stepped down and then off; and 34.8% stepped down only), suggesting the importance of informing patients about RAHS before de-prescription. Gronevalt et al. [54] conducted an RCT in Brazil, recruiting 45 patients with 32 months of median continuous PPI use. They reported the effectiveness of a 2-month course of spirulina platensis, a dietary supplement made from blue-green algae, to attenuate rebound dyspepsia (55.6% in spirulina platensis versus 88% in placebo) but not reflux symptoms (72% in spirulina platensis versus 76% in placebo) after PPI discontinuation.

Although there are no uniform methods to de-prescribe PPIs, some clinical practice guidelines (CPGs) on this issue have been proposed or updated recently. CPGs from the American Gastroenterological Association [55] state that “When de-prescribing PPIs, either dose tapering or abrupt discontinuation can be considered”, based on the fact that this effectiveness is still controversial. In clinical practice guidelines from Canada [56], when the discontinuation of PPIs is attempted, monitoring for symptom recurrence and managing symptoms with on-demand PPIs, stepping down to H2RA therapy, other over-the-counter agents, or nonpharmacologic approaches are recommended, taking into consideration the possibility of rebound.

## 2. Discussion

Although acid rebound has been demonstrated by well-designed physiological measurements [13,14], the results were generally met with skepticism. Several researchers doubted the clinical relevance of this “physiological” acid rebound. However, two well-designed landmark studies with robust endpoints and good methodology solved this issue [15,16]. These double-blind RCTs demonstrated symptoms of RAHS in healthy volunteers without a history of upper GI symptoms after withdrawal of PPI therapy but not after stopping the placebo. Results from these two studies were remarkably similar and showed that developed symptoms in previously asymptomatic healthy controls were able to prove that these symptoms were newly caused by PPI withdrawal. However, as described above, direct measurements of gastric acid secretion were not conducted in these studies, whereas the results were compatible with the role of RAHS in the symptoms of PPI withdrawal. This is why the clinical implications of RAHS after the discontinuation of PPIs are still unclear. To solve this issue, as an ideal study design, gastric acid secretion capacity (s-gastrin and p-CgA), gastric acid secretion (pH in the stomach), and acid reflux in the esophagus (pH in the esophagus) should be measured before PPI treatment, at the end of treatment (e.g., week 4), at an early (e.g., week 5) timepoint post withdrawal, at a middle (e.g., week 6) timepoint, and at a late (e.g., week 8) timepoint to correlate the change in those three factors. Furthermore, most of the previous scientific studies were designed with a relatively small sample size and/or under relatively short follow-up periods. Larger-sized studies with longer follow-up periods are important in the future to increase the knowledge in this field. However, these are challenging studies to perform and would probably need to be initiated by researchers in this field.

RAHS is important in clinical settings because it may lead to the reuptake of PPIs as symptoms are thought to originate from an underlying acid-related disorder, such as recurrent GERD, instead of being a temporary consequence of withdrawal therapy. This may lead to continuous prescriptions of PPIs. To avoid continuing unnecessary PPI use, it is essential for clinicians to understand RAHS and inform patients under long-term PPI treatment about the possibility of transient upper GI symptoms due to RAHS before discontinuing PPIs.

Studies on PPIs in patients with GERD found no signs of symptoms caused by acid rebound. [17,22,26]. However, a one-week duration of PPI therapy [22], a two-week duration of PPI therapy with a low dose of PPIs (omeprazole 20 mg) [26], or a low dose of PPIs on demand (lansoprazole: mean of 15.1 mg/day) [17] were adopted in these studies. These studies may be too short and/or use doses of PPIs that are too low to capture the potential symptoms related to RAHS. Another reason that the role of RAHS in the symptoms in patients with GERD has not been established may be because of the complexity of the assessment: differentiating the symptoms of rebound hypersecretion from symptoms of recurrent GERD is challenging. Peura et al. analyzed reflux symptoms in 294 patients during a 1-week follow-up period after the discontinuation of short-term PPI use (esomeprazole 20 mg for two weeks) using pooled data from two RCTs (290 placebo subjects). They concluded that lower baseline heartburn frequency and heartburn resolution during the last seven days of treatment were associated with a greater likelihood of heartburn resolution during the seven-day follow-up [57]. Thus, it is hard to determine whether the observed heartburn was caused by RAHS or not. No well-designed study with adequate methodology has been undertaken to study the clinical relevance of RAHS in patients with GERD.

If acid rebound occurs in patients with GERD as in healthy volunteers [15,16], it is conceivable that the contribution of RAHS to symptoms should be stronger for the patients with GERD as these patients frequently have visceral hypersensitivity or risk factors for reflux than healthy controls. Therefore, it may be more difficult for GERD patients to discontinue PPIs. In a study on the effects of the discontinuation of PPIs on long-term therapy (mean duration of 5 years), 73% of the patients failed to discontinue PPI therapy, which might have been driven by underlying GERD since 78% of the patients had symptoms indicating GERD at the baseline [38]. Furthermore, a recent study using ambulatory reflux monitoring demonstrated that the strongest predictor of PPI discontinuation was an acid exposure time of 4.0% on reflux monitoring [58]. Therefore, ingenious approaches are needed to manage those patients with symptoms at the baseline.

The other risk factors for developing RAHS in patients (age, gender, body mass index, hiatal hernia, etc.) are still largely unknown. The risk factors of the PPIs leading to RAHS, such as the minimum dose and time period to cause RAHS, are not fully understood. The findings of these factors as risk stratification would contribute to clinical practice. Further research is needed to determine the optimal method of PPI use and add preventive drugs after PPI withdrawal to reduce RAHS and achieve de-prescription.

## 3. Conclusions

This review summarized the current knowledge, its limitations, and future directions in the field of RAHS. Although the mechanism of RAS has been well established, clinical implications are still debatable based on the methodological weakness of the literature. Additionally, there is a lack of knowledge of risk stratification. Further research is needed to manage RAHS appropriately.

## Figures and Tables

**Table 1:** Summary of methods and outcome of the studies investigating RAHS.

Authors and Year	Type and Dose	Study Design	Participants	Measurements	Duration	Follow-Up	Outcome of RAHS	Country
**Physiological RAHS**								
Waldum et al., 1996 [13]	Omeprazole 40 mg/d	Pre-post study	9 patients with RE	Acid output Gastrin CgA	90 days	14 days	+	Norway
Gillen et al., 1999 [14]	Omeprazole 40 mg/d	Pre-post study	21 healthy volunteers (12 Hp-negative and 9 Hp-positive)	Acid output Intragastric pH Gastrin	56 days	15 days	+	UK
Fossmark et al., 2005 [23]	Omeprazole 40 mg/d, Esomeprazole 40 mg/d, or Lansoprazole 30 mg/d	Pre-post study	7 patients with RE (waiting for anti-reflux surgery)	Acid output Gastrin CgA	More than 1 year	182 days	+	Norway
Orr et al., 1995 [25]	Omeprazole 20 mg/d or Ranitidine 300 mg/d	Pre-post study	20 patients with GERD	24-h ambulatory esophageal pH	7 days 7 days	3 days 1 day	-	USA
**Symptomatic RAHS**								
Reimer et al., 2009 [16]	Esomeprazole 40 mg/d	Double-blind RCT	120 health volunteers	GSRS Gastrin CgA	56 days	28 days	+	Denmark
Niklasson et al., 2010 [15]	Pantoprazole 40 mg/d	Double-blind RCT	48 Hp-negative healthy volunteers	GDS Gastrin CgA	28 days	42 days	+	Sweden
Tanaka et al., 2023 [18]	PPI (Not unified) or VPZ	Pre-post study in multicenter setting	92 patients with RE	FSSG GSRS HADS Gastrin	More than 1 month	28 days	+	Japan
Farup et al., 2001 [22]	Lansoprazole 60 mg/d	Double-blind RCT with crossover design	62 patients with GERD	Symptom-related score	5 days	9 days	-	Norway
Wada et al., 2009 [33]	Rabeprazole 20 mg/d	Double-blind RCT	39 Hp-eradicated patients with severe gastric atrophy	QUEST score Endoscopy	56 days	112 days	-	Japan
Peura et al., 2016 [26]	Esomeprazole 20 mg/d	Double-blind RCT	1275 patients with heartburn	Symptom-related score	14 days	7 days	-	USA
Boyce et al., 2017 [24]	Esomeprazole 40 mg/d	Double-blind RCT	42 Hp-negative healthy subjects	Symptom-related score Gastrin CgA	28 days	14 days	-	UK
Juul-Hansen et al., 2011 [17]	Lansoprazole 15 mg/d On-demand (Max 60 mg/d, median 15.1 mg/d)	Pre-post study	26 patients with endoscopy-negative reflux disease	MGDQ Gastrin CgA 24-h esophageal pH monitoring	6 months	14 days	-	Norway

**Abbreviation:** RAHS—rebound acid hypersecretion, CgA—chromogranin, Hp—*Helicobacter pylori*, RE—reflux esophagitis, GERD—gastroesophageal reflux disease, RCT—randomized controlled trials, GSRS—Gastrointestinal Symptom Rating Scale, GDS—Glasgow dyspepsia score, VPZ—vonoprazan, FSSG—Frequency Scale for the Symptoms of GERD, HADS—Hospital Anxiety and Depression Scale, QUEST—questionnaire for the diagnosis of reflux esophagitis, and MGDQ—Modified Glasgow Dyspepsia Questionnaire.
